# Can Ebola virus become endemic in the human population?

**DOI:** 10.1007/s13238-015-0231-8

**Published:** 2015-12-16

**Authors:** Gary Wong, George F. Gao, Xiangguo Qiu

**Affiliations:** Special Pathogens Program, National Microbiology Laboratory, Public Health Agency of Canada, Winnipeg, MB Canada; Department of Medical Microbiology, University of Manitoba, Winnipeg, MB Canada; CAS Key Laboratory of Pathogenic Microbiology and Immunology, Institute of Microbiology, Chinese Academy of Sciences, Beijing, 100101 China; National Institute for Viral Disease Control and Prevention, Chinese Center for Disease Control and Prevention, Beijing, 100052 China; Office of Director-General, Chinese Center for Disease Control and Prevention, Beijing, 102206 China

The 2014–15 Ebola virus (EBOV) outbreak, originally reported during March 2014 in the Western African nation of Guinea, has shown itself to be resistant to traditional containment methods, with over 28,000 infections and 11,000 deaths over 18 months. Recently, news that a Scottish nurse had relapsed to EBOV disease with neurological symptoms at 10 months after recovery have astonished experts. The prolonged nature of the outbreak has led to questions whether EBOV can become endemic in the human population, an undesirable outcome due to the large amount of resources required to keep this virus under control. In this commentary, we discuss aspects EBOV disease with those caused by pathogens considered endemic in humans, as well as factors which may contribute to sustained EBOV transmission in humans.

Ebola virus (EBOV), of the genus *Ebolavirus* and family *Filoviridae*, was historically regarded as a re-emerging pathogen, in which natural infections of humans are sporadic and unpredictable, often with several years passing by before the emergence of a new case. EBOV infection is associated with high case fatality rates (CFR) in humans (up to 90%), but past disease outbreaks due to EBOV lasted only several months on average, and fatal cases numbered at most in the hundreds (CDC.gov, 2014). As such, EBOV was considered a minor public health threat in Africa at the time. Perceptions of this virus changed when the largest documented EBOV outbreak, first reported during March 2014, ran rampant through the Western African nations of Guinea, Sierra Leone and Liberia, causing over 28,000 infections and 11,000 deaths over the past 18 months (WHO.int, 2015a).

**Figure 1:**
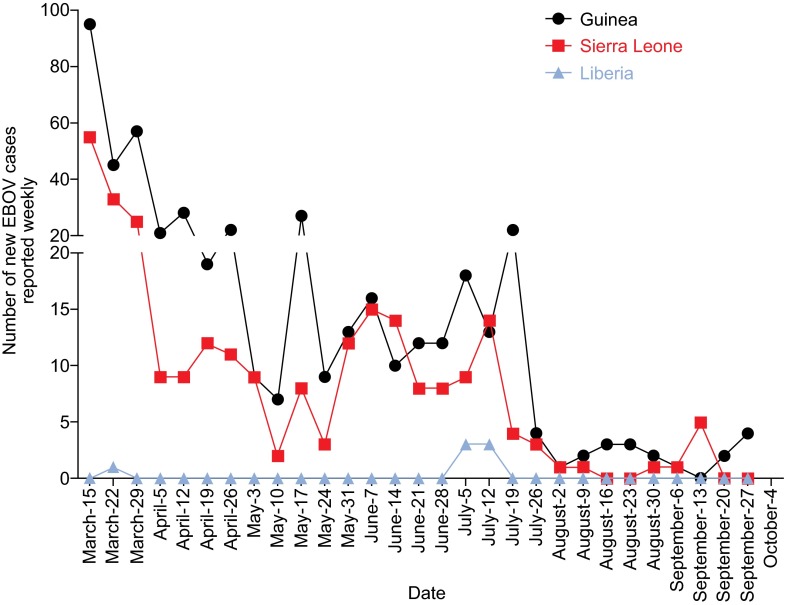
**Numbers of new EBOV infections per week in Guinea, Sierra Leone and Liberia**. Numbers are reported by the World Health Organization, and the dates are from March 15th to September 27th, 2015

Continued efforts by the local and international communities are having an observable effect in mostly confining EBOV transmission within the countries mentioned above, and the numbers of new weekly cases have been declining from a peak of over 700 in mid-September 2014 (WHO.int, 2015a). Since April 5th, 2015, the numbers of new cases reported weekly have fluctuated between 9 and 35, and this number has been less than 10 since July 26th, 2015 (Fig. 1). However, getting to zero cases overall has proven to be a challenge and of the three most heavily affected countries, only Liberia has been declared EBOV-free, on September 3rd, 2015 (WHO.int, 2015b). During this prolonged outbreak, some experts have questioned whether EBOV can become endemic within a human population (WHO Ebola Response Team, 2014) (NPR.org, 2014), a situation characterized by low levels of sustained virus transmission between humans, and the potential for a new outbreak to occur if this transmission is not successfully halted.

Can EBOV become endemic in the human population? If we contemplate other pathogens that are considered endemic within humans, such as Hepatitis B virus (HBV), Varicella Zoster virus (VZV) and human immunodeficiency virus (HIV), patients exposed to these viruses very rarely display major, specific symptoms early in the course of infection. In many cases the patients themselves do not know that they have been infected, and thus unknowingly transmit the virus to other susceptible hosts. In contrast, EBOV infection is typified by general, flu-like symptoms that rapidly progresses to severe hemorrhagic fever and multiple-organ failure within between 7–14 days, accompanied by very visible signs of disease such as rash (Feldmann et al., 2013). Patients are not known to be contagious during the incubation period (WHO.int, 2014), and the virus is primarily transmitted during advanced and terminal EBOV disease (Dowell et al., 1999). While asymptomatic infections with EBOV in humans have been documented on previous occasions (Baxter, 2000) (Becquart et al., 2010), there is no evidence thus far that asymptomatic humans can shed live virus. These factors prevent “silent” EBOV spread within an unsuspecting population.

On the other hand, live virus has been found in the semen of convalescent survivors at least 3 months after infection (Rowe et al., 1999) (Rodriguez et al., 1999). A Liberian female, who likely contracted EBOV after unprotected sex with a man that had recently recovered from infection (CDC.gov, 2015), showed that some EBOV survivors may still be contagious after recovery from the disease. During the 2014–15 outbreak, live EBOV has also been isolated from the ocular fluids of a physician who had survived infection, 9 weeks after the clearance of viremia (Varkey et al., 2015). The relapse of a Scottish nurse that had survived EBOV infection 10 months previously (Promedmail.org, 2015) suggests that supplementary diagnostics tests, in addition to viremia quantification by reverse transcription-quantitative polymerase chain reaction (RT-qPCR), may be needed to confirm the elimination of EBOV in convalescent survivors. Heightened awareness regarding the possibility of recovering survivors harbouring live EBOV is necessary, but due to the lower amounts of virus present within convalescent patients compared to those with advanced EBOV disease, it is unlikely that survivors play a major role with regards to EBOV propagation in humans.

From an evolutionary standpoint, EBOV does not have a significantly higher mutation rate (1.23 × 10^−3^ substitutions per site per year (Tong et al., 2015)) compared to other RNA viruses (Jenkins et al., 2002), and to date there are only five known member species of *Ebolavirus*. Published studies have also shown that EBOV is not significantly evolving, despite undergoing extensive passaging in humans during the 2014–15 outbreaks (Hoenen et al., 2015) (Tong et al., 2015). Therefore, the evidence at hand does not support the virus becoming endemic amongst the human population, and current response measures, which have steadily lowered the number of new weekly infections over time, should be sufficient in eventually ending the prolonged EBOV outbreak in Western Africa. However, the existence of possible “super-spreaders” of disease (Wong et al., 2015), and the relapse of Scottish nurse to EBOV disease is a reminder that close surveillance for any potential reoccurrence of EBOV disease in the human population is needed.
